# Genetic analysis of an elite super-hybrid rice parent using high-density SNP markers

**DOI:** 10.1186/1939-8433-6-21

**Published:** 2013-08-15

**Authors:** Meijuan Duan, Zhizhong Sun, Liping Shu, Yanning Tan, Dong Yu, Xuewu Sun, Ruifen Liu, Yujie Li, Siyu Gong, Dingyang Yuan

**Affiliations:** State Key Laboratory of Hybrid Rice, Hunan Hybrid Rice Research Center, Changsha, 410125 China; Hunan Academy of Agricultural Sciences, Changsha, 410125 China; Hunan Agricultural University, Changsha, 410125 China; Long Ping Branch of Graduate School of Central South University, Changsha, 410125 China; Beijing Genomics Insititute (BGI), Shenzhen, 518083 China; Guangdong Provincial Key Laboratory of Crop Genetic Resources Research and Application (NO.2011A091000047), Shenzhen, 518083 China

**Keywords:** Giant spike rice, Heterosis, MSG, Yield components, QTL mapping, Effect analysis

## Abstract

**Background:**

With an increasing world population and a gradual decline in the amount of arable land, food security remains a global challenge. Continued increases in rice yield will be required to break through the barriers to grain output. In order to transition from hybrid rice to super-hybrid rice, breeding demands cannot be addressed through traditional heterosis. Therefore, it is necessary to incorporate high yield loci from other rice genetic groups and to scientifically utilize intersubspecific heterosis in breeding lines. In this study, 781 lines from a segregating F_2_ population constructed by crossing the *indica* variety, “Giant Spike Rice” R1128 as trait donor with the *japonica* cultivar ‘Nipponbare’, were re-sequenced using high-throughout multiplexed shotgun genotyping (MSG) technology. In combination with high-density single nucleotide polymorphisms, quantitative trait locus (QTL) mapping and genetic effect analysis were performed for five yield factors (spikelet number per panicle, primary branches per panicle, secondary branches per panicle, plant height, and panicle length) to explore the genetic mechanisms underlying the formation of the giant panicle of R1128. Also, they were preformed to locate new high-yielding rice genetic intervals, providing data for super-high-yielding rice breeding.

**Results:**

QTL mapping and genetic effect analysis for five yield factors in the population gave the following results: 49 QTLs for the five yield factors were distributed on 11 of 12 chromosomes. The super-hybrid line R1128 carries multiple major genes for good traits, including *Sd1* for plant height, *Hd1* and *Ehd1* for heading date, *Gn1a* for spikelet number and *IPA1* for ideal plant shape. These genes accounted for 44.3%, 21.9%, 6.2%, 12.9% and 10.6% of the phenotypic variation in the individual traits. Six novel QTLs, *qph1-2, qph9-1, qpl12-1, qgn3-1, qgn11-1* and *qsbn11-1* are reported here for the first time.

**Conclusions:**

High-throughout sequencing technology makes it convenient to study rice genomics and makes the QTL/gene mapping direct, efficient, and more reliable. The genetic regions discovered in this study will be valuable for breeding in rice varieties because of the diverse genetic backgrounds of the rice.

**Electronic supplementary material:**

The online version of this article (doi:10.1186/1939-8433-6-21) contains supplementary material, which is available to authorized users.

## Background

Rice (*Oryza sativa L.*) is the world’s most important cereal crop and is the staple food for more than half of the world’s population (Mclean et al.^2002^). China has pioneered the advantages of heterosis in promoting the successful use of hybrid rice which has resulted in a steady increase in annual grain production from 0.35 billion t to 0.5 billion t. Per capita annual grain consumption in China is up to 0.4 t. This progress has reversed the fundamental problem of chronic food shortages and realized the basic coordination of grain supply and demand (Deng et al.^2010^). However, with an increasing world population and gradually deteriorating environment, food security has become a major challenge around the world, especially in Asia and Africa (Godfray et al.^2010^; Mcnally et al.^2009^; Sasson^2012^). Great cooperation and dedication is needed to break through the yield barrier by increasing the rice yield per unit area. Since the mid-1990s, a team of scientists led by Yuan Longping, an academic, has achieved 10.5, 12.0 and 13.5 t/ha by in super-hybrid rice and is approaching the target yield of 15.0 t/ha. Thus, hybrid rice has now reached the super-hybrid breeding stage. It is difficult to meet the demands of super-high yield by depending on heterosis. A significant breakthrough in super-hybrid rice breeding can be realized through modification of morphological traits in the super-hybrid parent and the scientific utilization of heterosis (Chen^2007^; Chen et al.^2010^).

Breeding practices have demonstrated that the discovery of specific germplasm and innovation in breeding materials are critical to successful breakthroughs in the development of super-hybrid rice (Cheng^2000^). Exploiting heterosis between rice varieties is the main strategy that is currently used in hybrid rice breeding. However, similar genetic backgrounds and reduced genetic diversity cause difficulties in restorer line breeding in hybrid rice combination for drastically improving the heterotic effect and increasing hybrid rice yield. This situation does not mean that the importance of heterosis in rice is reduced, but rather the difficulty in using heterosis has increased. Simply relying on heterosis between varieties cannot address the needs of super-hybrid rice breeding. Therefore, it is necessary to pursue a higher level of heterosis by using the genetic differences that exist in more distantly-related rice germplasm (subspecies, interspecific and intergeneric) in order to break through the bottleneck in traditional rice heterotic hybrid approaches, to maximally exploit the interaction potential of rice yield genes (additive, dominance, over-dominance and epistasis). This can also be used to help breed super-hybrid rice parental lines, and to accelerate the steps required for super-hybrid rice breeding (Deng et al.^2010^).

Rice yield is essentially dependent on four primary factors: (1) number of spikelets per panicle, (2) grain weight, (3) grain filling, and (4) the number of effective panicles. There are also some secondary factors such as plant height and panicle length that effect the rice yield. Rice yield is a complex quantitative trait, and its increase is dependant on synergy of all these factors (Gao et al.^2011^). Breeding of dwarf rice in China began in the late 1950s and dwarfing in the basic production indica rice was finished in the late 1960s. Subsequently, breeders rebuilt the plant and leaf shapes using the *Sd-1* gene and explored ways to further increase rice potential yield through giant panicles and grains (Liu et al.2012b). Over the past 20 years, breeders have focused on increasing the potential yield in rice by increasing the giant panicle type when modifying rice varieties. One study maintained that the potential yield of early rice in the Yangtze River Basin can be increased 15%–20% by reducing the number of panicles and increasing individual panicle weight (Zhu et al.^2007^). Constant genetic recombination (hybridization and selection) allows a new balance and coordination between the number of panicles per plant and spikelet number per panicle in rice to be attained, thus fulfilling an ideal high-yielding purpose. The optimal combination of modification for ideal plant shape and utilization of heterosis are an inevitable path for super-hybrid rice breeding. The molecular design of the “ideal plant architecture with giant panicle” and “erect super-hybrid rice with giant panicle” is the direction to be taken in future rice breeding efforts (Liu et al.2012b).

The super-hybrid rice parent R1128 is an optimal restorer line with giant panicles, which was created throughout the hybridization of distantly-related subspecies under the above-mentioned theoretical concept and was recognized as “Giant Spike Rice” by Yuan Longping. This line has prominent features of high grain number per panicle (up to a maximum of 980 under the high-yielding cultivation regime of high fertilizer and low density planting, far higher than the strong restorer lines 9311 and Minghui 63, which average 400–600), strong stem, high lodging resistance and high seed setting rate. R1128 also resolves the contradictions of giant panicle and lower seed setting rate in previous generations when a significant breakthrough was achieved in creating hybrid rice parents through wide crosses between subspecies.

With the recent advances in DNA sequencing technology and functional genomics, more species are resolved and more functional genes are mapped and cloned. In this study, 781 lines from the segregating indica-japonica F_2_ population (constructed by crossing the *indica* donor parent, “Giant Spike Rice” R1128 with the *japonica* cultivar Nippobare) were re-sequenced using high-throughput multiplexed shotgun genotyping (MSG) technology (Andolfatto et al.^2011^). In combination with high-density single nucleotide polymorphism (SNP) (Lander^1996^) molecular markers, quantitative trait locus (QTL) mapping and genetic effect analysis were performed on five yield related factors. Grain number per panicle, primary branches per panicle, secondary branches per panicle, plant height, and length of main panicle in order to investigate the genetic mechanism underlying the formation of the R1128 giant panicle and to identify and locate the new high-yielding rice gene locus to provide useful data for super-high yield rice breeding.

## Results

### Sequencing and SNP identification

We sequenced the cultivar R1128 using the Illumina Hiseq2000 platform, yielding about 6.17 G bases of raw data. The short reads were mapped back to the IRGSPv6 rice genome using SOAP2 (version 2.20). The genome coverage was about 87% and the effective mapping depth reached >16×. About 690, 720 SNPs, or 1.8 SNPs/kb, were identified between the parents using a strict analysis pipeline (Table 1).

**Table 1:** **Number of SNPs per chromosome in the R1128 X Nipponbare F**_**2**_ **population**

Chromosome	Number of homozygous SNPs between parental lines	Number of SNPs in population
Chr01	65950	8063
Chr02	76438	6471
Chr03	70935	6926
Chr04	46988	6067
Chr05	53788	4482
Chr06	60384	7884
Chr07	62164	6107
Chr08	55706	7454
Chr09	50857	5228
Chr10	44812	5387
Chr11	63131	6388
Chr12	39567	3872
Total	**690720**	**74329**

**Figure 1:**
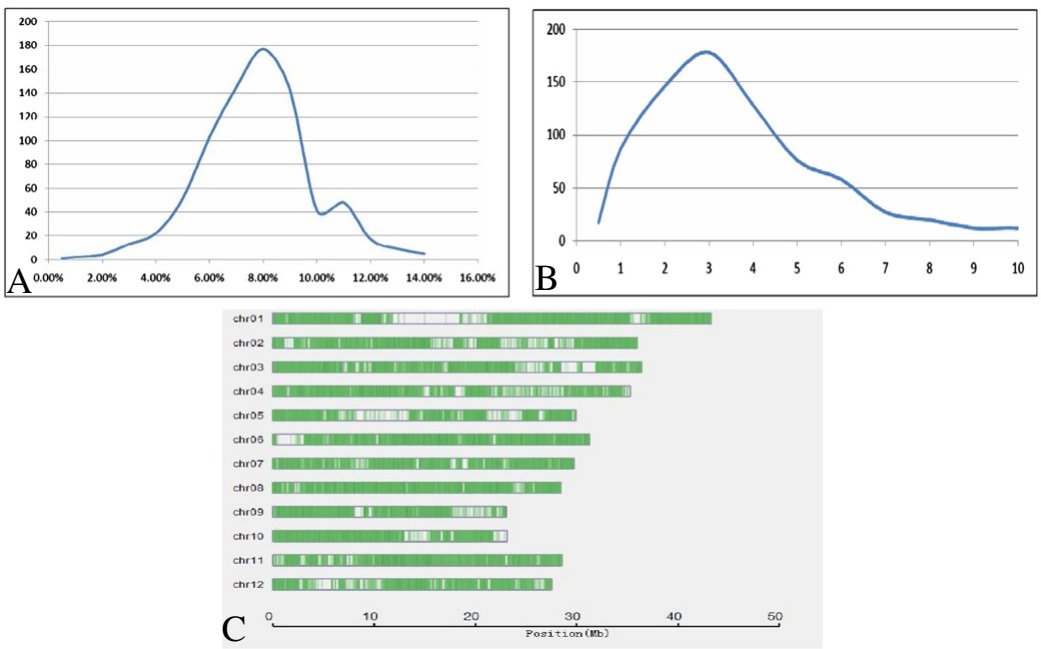
**Sequencing results for the F2 population. (A)** Population coverage distribution. The horizontal axis shows the coverage and the vertical axis represents the number of individuals. **(B)** Sequencing depth distribution. The horizontal axis shows the sequencing depth and the vertical axis represents the number of individuals. **(C)** SNP distribution on the 12 rice chromosomes.

The restriction enzyme fragments ranging from 400 bp to 600 bp for 781 F_2_ individuals were sequenced and generated a total of 107.96 Gbp of raw data, which is approximately 138.23 Mbp for each line (Figure 1B). The sequenced sites accounted for 8–12% of the whole genome and on average, the depth of each site was approximately two to six times greater in each individual (Figure 1A).

Population SNPs were filtered by the sites and were different between the two parents. The SNPs that were obviously due to noise were removed manually. A total of 74,329 SNPs or 1 SNP per 5 kb were detected for the F_2_, and the distribution of SNPs was even throughout the entire genome (Table 1, Figure 1C and Additional file1).

We compared these 74,329 SNPs to rice SNP database build by OryzaSNP Consortium. A total of 9,377 SNPs can be found in this SNP database and all of these SNPs differ from the reference genome allele. We add the SNPs information to this rice whole genome resequencing genotype data file (Additional file2).

### Recombination breakpoint determination and Bin map construction

In an F_2_ population, the breakpoints separate homozygous and heterozygous genotypes and also separate one homozygous genotype from the other. We determined the recombination breakpoints by checking the positions where genotypes change from one type to the other when placed along the chromosomes. A total of 22,594 breakpoints were identified for the 781 individuals, for an average of 29.39 per individual (Additional file3 and Additional file4).

After we determined the recombination breakpoints for each individual, we constructed a skeleton bin map. A total of 6,819 bins were detected for the 781 F_2_ progeny for the minimum 10 kb intervals (Table 2 and Additional file5). Each bin’s physical length ranged from 10.01 kb to 3.39 Mb, averaging 54.6 kb (Additional file6).

**Table 2:** **Number of bins per chromosome**

Chromosome	Number of bins
Chr01	979
Chr02	686
Chr03	700
Chr04	527
Chr05	541
Chr06	578
Chr07	603
Chr08	573
Chr09	364
Chr10	381
Chr11	517
Chr12	370
Total	6819

### Phenotypic variation and distribution

**Figure 2:**
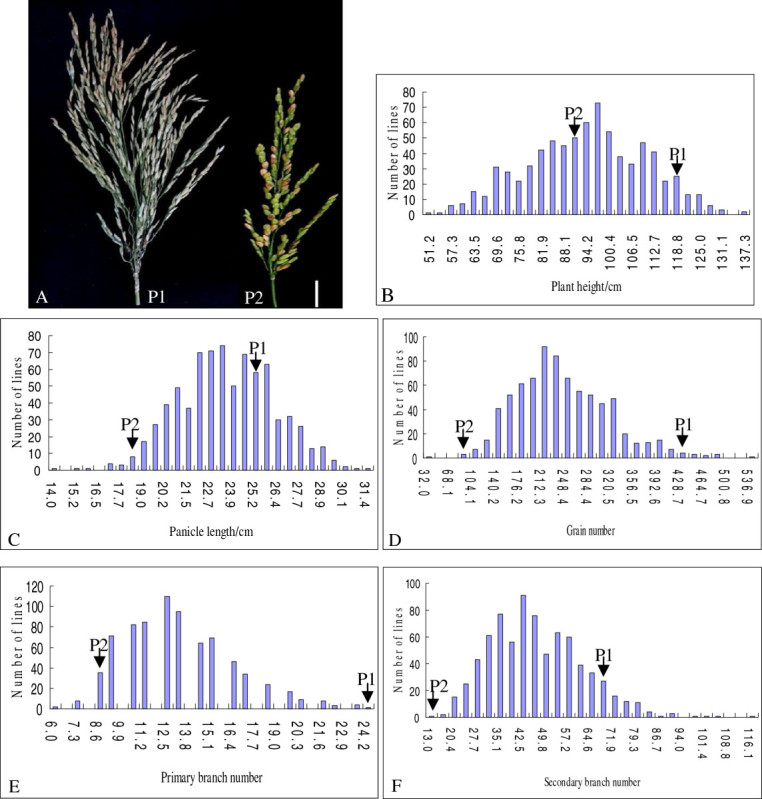
**Field performance comparison between parents and distribution of yield components in the F2 population. (A)** Panicle shape comparison between R1128 and Nipponbare. The P1 and P2 represent R1128 and Nipponbare respectively. Scale bar: 3 cm. **(B)** Plant height. **(C)** Panicle length. **(D)** Grain number per panicle. **(E)** Primary branch number. **(F)** Secondary branch number.

The five yield component traits, which included plant height (PH), panicle length (PL), grain number (GN), primary branch number (PBN) and secondary branch number (SBN), were investigated between R1128 and Nipponbare at Changsha in 2011. The GN of the super rice parent R1128 was nearly five times that of Nipponbare, reaching 438. Moreover, the increase in GN resulted from an apparent increase in the PBN and SBN (Figure 2). Extreme variations were also found in the yield components PH and PL. All trait values were significantly different at the 5% level between the two parents. The restorer line R1128 was distinctly different from the *japonica* rice line Nipponbare, and provided an abundant source of trait variation for population construction and QTL mapping.

Phenotypic values of the yield related traits PL, GN, PH, PBN and SBN were all found to be continuous with normal distribution and the values of population skewness and kurtosis were all less than 1. Bi-directionality was also observed for all traits and the values for the two parents were all within the range of the population. This indicates that yield component traits were quantitative and transgressive segregation could be generated from gene recombination. All characters in the population met the requirements for QTL mapping (Figure 2).

### QTL identification and effect calculations

In this study, five yield related traits including primary rachis branch, secondary rachis branch, plant height, grain number and main panicle length were examined with the bin map. Results with respect to trait QTLs identified are presented in Table 3.

**Table 3:** **QTL mapping and genetic effect analysis**

Trait	LOD threshold	Position; interval (Chromosome; cM)	QTL	Locus; size (peak bin; Kb)	Lod	Add	Dom	D/[A]	R^2^(%)	Included QTLs/genes
PH	3.2	1; 17.3-17.6	*qph1-1*	bin132; 14.6	2.35	−2.64	1.49	0.57	1.4	*ph1.1* (Marri et al.^2005^)
		1; 22.7-23.2	***qph1-2***	bin172; 13.2	2.57	−2.88	0.88	0.31	1.5	
		1; 131.0-131.5	*qph1-3*	bin807; 19.7	97.92	14.98	5.44	0.36	44.3	*sd-1* (Sasaki et al.^2002^)
	2.9	2; 87.5-87.7	*qph2-1*	bin1418; 34.0	2.98	−2.71	−2.65	−0.98	1.8	*Sn2a* (Tan et al.^1996^)
	3.1	3; 48.3-50.7	*qph3-1*	bin1846; 10.9	6.15	−4.31	−0.5	−0.12	3.6	*d88* (Gao et al.^2009^)
		3; 70.7-71.1	*qph3-2*	bin1943; 40.9	3.3	−3.29	0.17	0.05	2	*OsApx1* (Agrawal et al.^2003^)
	2.9	4; 121.9-122.4	*qph4-1*	bin2728; 106.0	2.83	−1.21	3.83	3.17	1.7	*qPH1-4-1* (Cui et al.^2004^)
	2.9	6; 26.6-27.1	*qph6-1*	bin3619; 57.6	19.37	−7.42	−2.58	−0.35	10.9	*qPH2-6-1* (Cui et al.^2004^); *ph6* (Xiao et al.^1996^)
		6; 58.1-58.4	*qph6-2*	bin3845; 78.0	7.73	−5.23	1.18	0.23	4.5	*qIN3-6* (Yamamoto et al.^2001^)
	2.8	9; 0.6-1.5	***qph9-1***	bin5195; 53.0	4.08	−3.49	2.01	0.58	2.4	
	2.6	10; 47.2-48.0	*qph10-1*	bin5796; 88.3	10.73	5.32	−2.89	−0.54	6.2	*OsCesA7* (Tanaka et al.^2003^)
	2.8	12; 66.8-68.0	*qph12-1*	bin6735; 107.2	3.58	−3.15	2.49	0.79	2.1	*nd1* (Li et al.2009a)
PL	3.1	1; 131.3-131.7	*qpl1-1*	bin808; 23.3	6.41	0.7	0.4	0.57	3.8	*qp1-1* (Hittalmani et al.^2002^)*; p11.1* (Thomson et al.^2003^)
	3	2; 22.9-23.2	*qpl2-1*	bin1175; 25.2	5.04	0.63	−0.39	−0.62	3	*qph-2* (Ping et al.^2003^)
	2.9	3; 48.3-50.7	*qpl3-1*	bin1846; 10.9	8.38	−0.84	0.06	0.07	4.9	*d88* (Gao et al.^2009^)
		3; 70.7-71.1	*qpl3-2*	bin1943; 40.9	6.48	−0.74	−0.07	−0.09	3.8	*OsApx1* (Agrawal et al.^2003^)
	2.9	4; 77.7-78.2	*qpl4-1*	bin2570; 20.3	3.19	0.47	0.33	0.7	1.9	*pl4* (Zhuang et al.^1997^)
	2.9	6; 26.6-27.1	*qpl6-1*	bin3619; 57.6	13.37	−1.04	−0.32	−0.31	7.7	*qPH2-6-1* (Cui et al.^2004^); *ph6* (Xiao et al.^1996^)
		6; 52.7-53.4	*qpl6-2*	bin3807; 13.1	20.28	−1.35	0.24	0.18	11.5	*OsIAA23* (Jun et al.^2011^); *ph6* (Xiao et al.^1996^)
	2.9	8; 87.1-87.4	*qpl8-1*	bin5156; 37.6	9.7	0.89	0.11	0.12	5.7	*IPA1* (Jiao et al.^2010^)
	2.6	10; 41.4-41.6	*qpl10-1*	bin5745; 268.2	5.1	0.59	−0.45	−0.76	3	*brd2* (Hong et al.^2005^)
	2.8	12; 68.8-74.3	***qpl12-1***	bin6738; 11.9	3.14	−0.46	0.47	1.03	1.9	
GN	3.1	1; 18.4-18.7	*qgn1-1*	bin137; 13.4	23.01	−37.07	−5.39	−0.15	12.9	*Gn1a* (Ashikari et al.^2005^)
		1; 127.5-128.0	*qgn1-2*	bin784; 14.5	8.74	22.73	9.1	0.4	5.1	*qLVBTS1-1* (Cui et al.^2003^)
	3	2; 86.0-86.7	*qgn2-1*	bin1394; 189.6	3.29	−15.64	−2.96	−0.19	2	*qPN2* (Yuan et al.^2003^)
	3.2	3; 66.6-67.0	***qgn3-1***	bin1929; 16.9	2.36	12.6	13.61	1.08	1.4	
		3; 137.1-137.5	*qgn3-2*	bin2208; 23.2	2.2	12.05	1.01	0.08	1.3	*ps3* (Redona and Mackill^1998^)
	2.9	6; 9.7-10.2	*qgn6-1*	bin3519; 70.9	3.76	−16.05	8.97	0.56	2.2	*TNSP6* (Zhuang et al.^2001^)
		6; 26.2-26.5	*qgn6-2*	bin3612; 41.0	16.36	−32.9	−1.59	−0.05	9.4	*tns6* (Lin et al.^1995^)
		6; 32.9-33.3	*qgn6-3*	bin3660; 135.5	15.62	−31.37	−5.62	−0.18	8.9	*OsJMT1*(Kim et al.^2009^)
		6; 36.5-36.8	*qgn6-4*	bin3685; 64.2	15.7	−31.35	−5.72	−0.18	9	*gp6* (Hua et al.^2002^)
	2.7	8; 67.1-67.8	*qgn8-1*	bin5104; 14.8	18.73	−33.28	15.65	0.47	10.6	*IPA1* (Jiao et al.^2010^)
	2.7	11; 26.1-26.5	***qgn11-1***	bin6104; 17.2	3.01	−14.04	−0.43	−0.03	1.8	
PBN	3.2	1; 18.4-18.7	*qpbn1-1*	bin137; 13.4	5.55	−0.82	−0.12	−0.15	3.3	*Gn1a* (Ashikari et al.^2005^)
	3.1	2; 79.8-80.4	*qpbn2-1*	bin1364; 16.6	6.02	−0.85	−0.17	−0.2	3.6	*np2.2* (Marri et al.^2005^)
	2.9	6; 9.4-9.9	*qpbn6-1*	bin3517; 37.4	12.41	−1.3	0.04	0.03	7.2	*qNPB6-1*(Cui et al.^2002^)
		6; 25.5-26.0	*qpbn6-2*	bin3607; 95.9	41.17	−2.02	−0.96	−0.48	21.9	*Hd1* (Yano et al.^2000^)
		6; 36.5-36.8	*qpbn6-3*	bin3685; 64.2	31.76	−1.8	−0.8	−0.44	17.4	*gp6* (Hua et al.^2002^)
	2.9	7; 65.3-65.7	*qpbn7-1*	bin4418; 71.0	2.55	−0.39	0.57	1.48	1.5	*OsFOR1* (Jang et al.^2003^)
	2.8	8; 68.0-68.4	*qpbn8-1*	bin5107; 45.0	14.26	−1.33	0.45	0.34	8.2	*IPA1* (Jiao et al.^2010^)
	2.6	10; 47.9-48.6	*qpbn10-1*	bin5798; 39.8	10.57	0.83	−1.08	−1.3	6.2	*Ehd1* (Doi et al.^2004^)
SBN	3.1	1; 18.4-18.7	*qsbn1-1*	bin137; 13.4	25.31	−7.98	−0.9	−0.11	14.1	*Gn1a* (Ashikari et al.^2005^)
		1; 127.5-128.0	*qsbn1-2*	bin784; 14.5	8.72	4.62	2.03	0.44	5.1	*qNSB1-1* (Cui et al.^2002^)
	3.1	3; 135.2-135.5	*qsbn3-1*	bin2186; 373.4	2.95	2.8	1.2	0.43	1.8	*ps3* (Redona and Mackill^1998^)
	2.9	6; 26.2-26.5	*qsbn6-1*	bin3614; 107.2	10.21	−5.46	0.9	0.16	5.9	*tns6* (Lin et al.^1995^)
		6; 29.9-31.2	*qsbn6-2*	bin3649; 29.5	11.38	−5.59	−0.49	−0.09	6.6	*qSPN-6* (He et al.^2001^)
		6; 36.5-36.8	*qsbn6-3*	bin3685; 64.2	10.76	−5.44	−0.53	−0.1	6.3	*gp6* (Hua et al.^2002^)
	2.8	8; 67.1-67.8	*qsbn8-1*	bin5104; 14.8	22.34	−7.55	2.62	0.35	12.6	*IPA1* (Jiao et al.^2010^)
	2.9	11; 26.1-26.5	***qsbn11-1***	bin6104; 17.2	2.4	−2.57	−0.11	−0.04	1.4	

Note: LOD threshold was calculated using Mapqtl software with 1000 iterations of the arrangement test; R^2^: phenotypic variation rate, D/[A]: additive (d/a = 0-0.2), partially dominant (d/a = 0.2-0.8), dominant (d/a = 0.81-1.2), overdominant (d/a > 1.2); The bold and italic type of QTLs were represented new one.

### Plant height(PH)

Plant height in the R1128 X Nipponbare F_2_ population was influenced by 12 genomic regions on eight chromosomes. The phenotypic effect (R^2^) variance explained by these QTLs ranged between 1.4% (*qph1-1*) and 44.3% (*qph1-3*). Out of these QTLs, only *qph1-3* and *qph10-1* had positive additive effects with values of 14.98 and 5.32, which were contributed from the R1128 alleles. *Qph1-3* had the highest LOD score (97.92) and the largest percentage of phenotypic variation (44.3%), followed by *qph6-1* (LOD = 19.37 and R^2^ = 10.9%). Phenotypic variations contributed from Nipponbare alleles were also noticed for 10 QTLs (*qph1-1*, *qph1-2*, *qph2-1*, *qph3-1*, *qph3-2*, *qph4-1*, *qph6-1*, *qph6-2*, *qph9-1* and *qph12-1*), showing negative additive effects ranging from −1.21 to −7.42 and explaining 1.4% to 10.9% of the phenotypic variation in plant height. Viewed from the standpoint of gene interaction (d/a), two QTLs (*qph3-1* and *qph3-2*) manifested mainly additive effects and most of these 12 QTLs showed positive or negative partial dominance, which included *qph1-1*, *qph1-2, qph1-3, qph6-1*, *qph6-2*, *qph9-1, qph10-1* and *qph12-1.* We also observed that *qph2-1* and *qph4-1* were the only 2 QTLs showing negative dominance and positive over dominance, respectively.

### Panicle length(PL)

Panicle length, one of the most important of the yield related characters, was controlled by 10 QTLs that would be distributed on chromosomes 1, 2, 3, 4, 6, 8, 10 and 12. Among these, *qpl6-2* located at bin3807 had the highest LOD value (20.28) and morphological variation score (11.5%) and displayed a negative additive effect mainly with the positive allele from Nipponbare. Considered from the standpoint of gene interaction, the other 3 QTLs (*qpl3-1, qpl3-2, and qpl8-1*) were all major with a negative or positive additive effect and explained 14.4% of the total phenotypic variation. *qpl12-1*, located in bin6738, had a positive overdominant effect alone, with a LOD score of 3.14 and R^2^ = 1.9%. The remaining five QTLs (*qpl1-1, qpl2-1, qpl14-1, qpl16-1 and qpl10-1*) that control the length of the main panicle show mainly positive or negative partial dominance effects; among these, *qpl6-1* has the highest LOD value (13.37) which explains 7.7% of the phenotypic variation, and the positive allele originated from the female parent Nipponbare.

### Grain number(GN)

Eleven QTLs associated with total GN per main panicle mapped to six different chromosomes in rice. Conclusions may be drawn that the five QTLs, *qgn1-1, qgn6-2, qgn6-3, qgn6-4* and *qgn8-1* have large LOD scores above 15. QTL *qgn1-1* has the highest LOD score of 23.01 and contribution rate of 12.9%. With the exception of *qgn8-1*, which has a partial dominant positive effect, the other four genes have additive effects, and the positive alleles originated in Nipponbare. For the remained six QTLs, the positive alleles of *qgn1-2, qgn3-1* and *qgn3-2* came from the male parent R1128. These QTL show a partial positive dominant effect, a positive overdominant effect, and a positive dominant effect, respectively and explain 7.8% of the total phenotypic variation. Additionally, three QTLs (*qgn2-1, qgn6-1, qgn11-1*) have additive, partial positive dominant, and additive effects, respectively. The positive alleles came from Nipponbare, and the total contribution rate was 6%.

### Primary branch number (PBN)

A total of eight QTLs associated with PBN were mapped in the F_2_ population and they were distributed on chromosomes 1, 2, 6, 7, 8 and 10. The QTLs controlling the PBN on chromosome 6 were up to three maximally (*qpbn6-1, qpbn6-2* and *qpbn6-3*); the highest LOD score was for *qpbn6-2* at 41.17, with a QTL contribution rate of 21.9%. The positive alleles were from the Nipponbare parent with a negative partial dominant effect; the other 2 QTLs have LOD scores of 12.41 and 31.76, respectively,. The positive alleles also came from Nipponbare with additive or partial negative dominant effects. The QTL *qpbn10-1* with LOD score 10.57 has a positive allele from the paternal line R1128 only and its contribution rate is 6.2% with a negative overdominance effect. For the remaining four QTLs, *qpbn1-1* and *qpbn2-1* had additive effects, and *qpbn7-1* and *qpbn8-1* had positive overdominant and positive partial dominant effects, respectively. The positive alleles of these four QTLs came from Nipponbare and had a contribution rate of 1.5–8.2%.

### Secondary branch number (SBN)

Eight QTLs, *qsbn1-1, qsbn1-2, qsbn3-1, qsbn6-1, qsbn6-2, qsbn6-3, qsbn8-1* and *qsbn11-1*, which are associated with SBN, mapped to five chromosomes (1, 3, 6, 8, 11). Their LOD values range from 2.40 to 25.31, with phenotypic contribution rates of 1.4%-14.1%. Three QTLs (*qsbn1-2, qsbn3-1, qsbn8-1*) have partial positive dominant effects. Among these, *qsbn1-2* and *qsbn3-1* possed positive alleles which came from R1128. The *qsbn8-1* allele came from Nipponbare; the other five QTLs (*qsbn1-1, qsbn6-1, qsbn6-2, qsbn6-3* and *qsbn11-1*) showed additive effects and their positive alleles were from Nipponbare.

## Discussion

### R1128 contribution and its value in rice breeding

The restorer line R1128 resulted from taking full advantage of wide crosses between rice subspecies. This line has excessive GN, high lodging resistance and strong combining ability, thereby coordinating the giant panicle and seed setting difficulties. This not only provides an important technical route for super-hybrid rice parent breeding, but also provides a basis to test heterosis theory.

In 2011, Liangyou 1128 (P88S/R1128), the super-hybrid rice combination bred from the super restorer line R1128 and two-line male sterile line P88S, was identified as single-cropping late rice (Xiangshen rice 2011024) in Hunan Province. This variety shows a high and stable yield, good plant shape, grain quality and high lodging resistance (the lodging resistance coefficient is up to 250 g or above at about 120 cm PH). In the years 2009–2010, the line was put to trial as seasonal late rice in Hunan Province. Its average yield was 8.52 t/ha, 6.19% higher yield than Shanyou 63; yield per day was 0.0672 t/ha, 0.0018 t/ha, higher than the control. In 2010, the super rice combination was tested in small area plots (0.12 ha) to test its high-yielding potential. After being harvested by experts, the yield was up to 14.47 t/ha (Liu et al.2012a). According to incomplete statistics, Liangyou 1128, the combination of super-hybrid rice, was grown promotionally over 66,666.67 hectares in 2011/2012 to lay a foundation for the stable and increasing production and income of farmers.

After attaining 13.89 t/ha in large plots last year and meeting the goal of the third stage ahead of time, a research team led by Yuan Longping advanced to the fourth stage of 15 t/ha of super-hybrid rice in large area plots. Three hybrid rice combinations: Y Liangyou 1128 (Y58S/R1128), Guangliangyou 1128 (Guangzhan 63-2S/R1128) and 4001S/R1128, were all using the super-hybrid rice restorer line R1128 as a parent and were made for the purpose of attaining 15 t/ha. It can be expected that the target of 15 t/ha can be achieved by using R1128 in combination under the technical guidelines of “elite seeds, correct method, suitable field and better ecology” proposed by Yuan Longping.

### MSG for identification of genetic variation

MSG (multiplexed shotgun genotyping) is one method of reduced-representation sequencing and has some significant advantages for genome-wide genetic marker discovery and genotyping. MSG requires only a set of bar-coded adapters and one ligation step, followed by fragment size selection. It costs much less than RAD (restriction site associated DNA) (Baird et al.^2008^) due to its simple procedure and reduced requirements for laboratory equipment. Library preparation for the 781 individuals required only four days, which shows the dramatic high-throughput potential of this method. Compared to arrays, MSG gives more efficient and evenly distributed genetic markers for genotyping. The fragment selection size ranges from 300 bp to 800 bp, and can be adjusted for different research materials and objectives. Sequencing a narrow size range of DNA fragments can get enough markers for highly divergent lines. In this study, about one-tenth of the total SNPs (74329/690720) detected by sequencing 400 bp–600 bp fragments showed high resolution for breakpoint determination and QTL mapping. For low divergence lines, a wider range fragments should be selected to get more informative markers. Other studies have shown that even divergence as low as 0.5% between parental lines allowed resolution of half of the recombination breakpoints to within 136 kb, which is sufficient for QTL studies involving genotyping of hundreds of individuals (Andolfatto et al.^2011^; Mackay^2001^). MSG reduces the genome complexity similar to other methods that are based on restriction enzyme digestion. As a result, data analysis is more efficient and can be done on computers with medium performance. The genotypes of all 781 individuals were determined in only one week, and appropriate individuals were then selected for the hybrid experiment.

### Verification and analysis of QTL mapping

**Figure 3:**
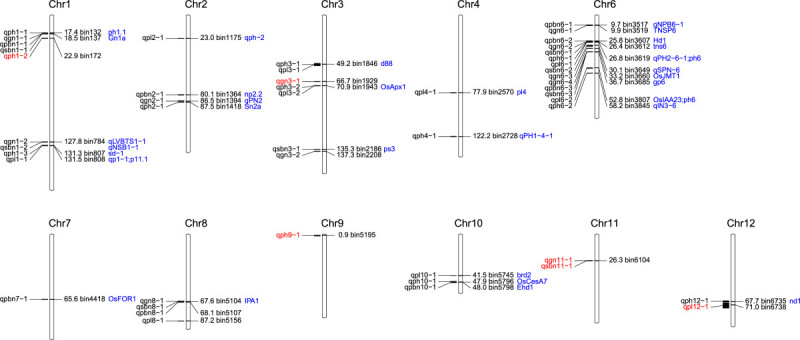
**Bin linkage chromosomal map showing locations of QTLs related to rice yield factors.** Chromosome numbers are indicated above and Bin names and genetic distance (cM) from the distal end of the short arm of each chromosome are shown. The letters marked with blue represent the previously known QTLs/genes and the opposite were the QTLs mapped in this research, besides the letters marked with red respresent the new QTLs for grain yield components.

Forty-nine QTLs for five key yield factors were mapped in the F2 population and were found to be distributed on all 12 chromosomes except for chromosome 5. Among these, 14 QTLs were mapped to chromosome 6 alone (Figure 3).

**Figure 4:**
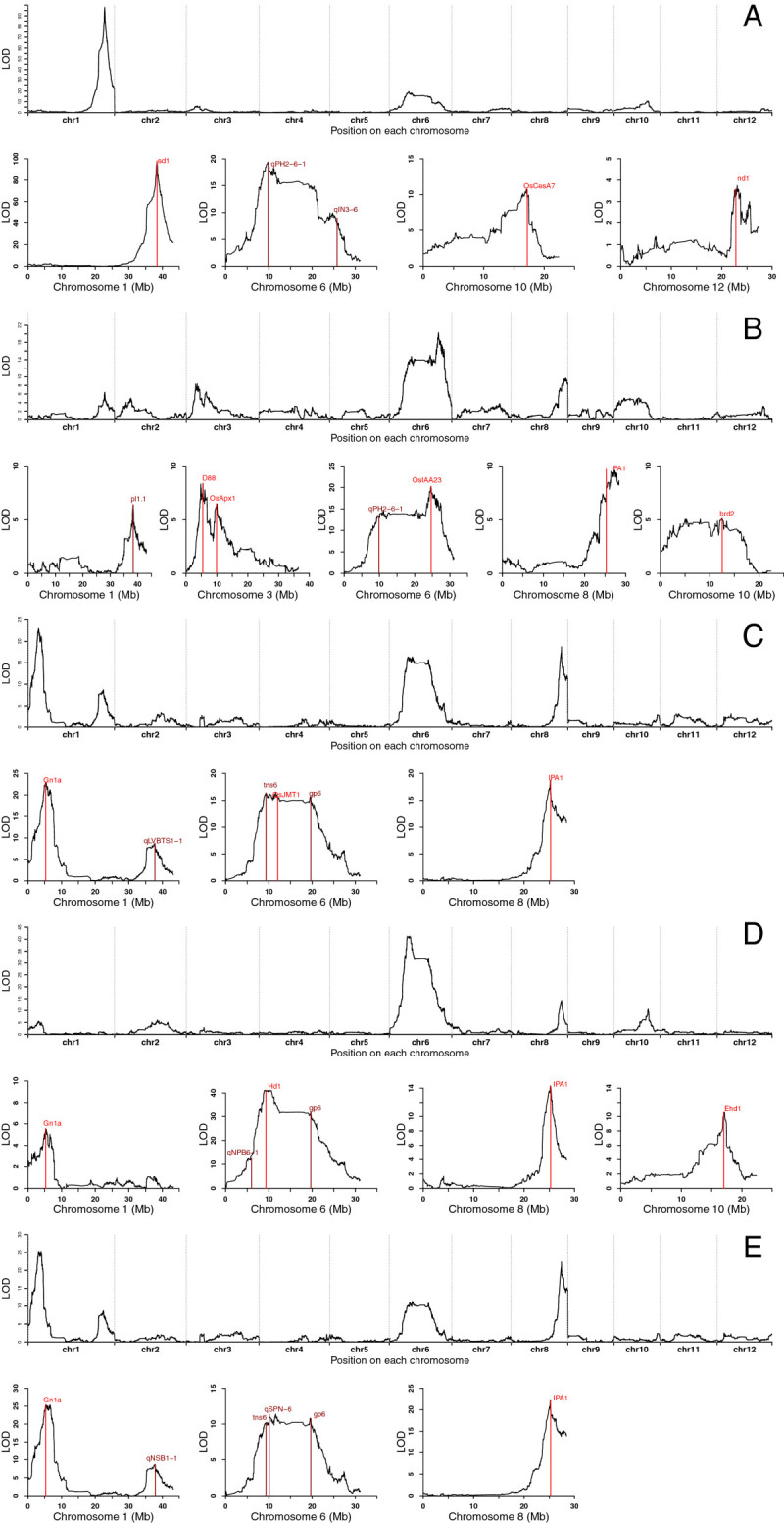
**Genome-wide scan in the F2 population and QTL trait mapping. (A)** Plant height. **(B)** Main panicle length. **(C)** Total grain number per panicle. **(D)** Primary branch number. **(E)** Secondary branch number.

Twelve associated genetic intervals were mapped for PH. In comparing QTLs defined in this study to previous research, *qph1-3*, with the highest peak value on chromosome 1, is located at the same locus as the rice “Green Revolution” gene *sd-1*(Sasaki et al.^2002^). LOD in the population reaches 97.92 with a contribution rate of 44.3% as shown in Figure 4A; *qph1-1* is located on the short arm of chromosome 1 within a 14.6 kb interval, just in the area of *ph1.1* and compared with the more finely mapped region by (Marri et al.^2005^). The study of (Zhao et al.^2009^) considered that inheritance of PH is commonly controlled by its ground internode length. Examples can be found in this study: *qph2-1* mapped here and the second internode length locus *Sn2a* mapped by (Tan et al.^1996^) are located in the same interval. Additionally, *qph6-2* and *qIN3-6* (Yamamoto et al.^2001^) on chromosome 6 are also mapped to the same interval. This indicates that these areas maybe have loci that control PH or internode length. The other two genetic intervals, *qph3-2* and *qph4-1*, were also reported in the previous studies. *Qph3-2* is covering the cytoplasm ascorbate peroxidase gene *OsApx1* exactly; (Agrawal et al.^2003^); *qph4-1* and *qPH1-4-1* are in the same region (Cui et al.^2004^); *qph3-1* located on the short arm of chromosome 3 is localized within 10.9 kb while the esterase gene *d88* cloned by (Gao et al.^2009^) is within this interval. Attention should be paid to the freauently-appearing high peak value on chromosome 6 where the *qph6-1* LOD score reached 19.37 at a contribution rate 10.9%. The locus is included within the interval between *qPH2-6-1* mapped by (Cui et al.^2004^) and *ph6* mapped by (Xiao et al.^1996^). The LOD scores of *qPH2-6-1and ph6* are 22.51 and 5.36, respectively, explaining the phenotypic variation rates of 38.4% and 12.1%. The above loci had some value in rice PH breeding. (Tanaka et al.^2003^) had studied and cloned the *OsCesA7* gene, which encoded a cellulose synthase catalytic subunit involved in the biosynthesis of the cellulose. *OsCesA7* after mutation can significantly reduce the cellulose content in the stalk, making the stem brittle and thin which would result in a dwarf plant. *Qph10-1* located on chromosome 10 (peak value LOD = 10.37) in this study, is close to the gene *OsCesA7*. It has been speculated that the variation is due to the different genetic background and mapping approaches, *etc.* (Zheng et al.^2003^). *Qph12-1* is targeted in the region which includes rice cellulose synthase gene *nd1* (Li et al.2009a). The minor QTL (*qph1-2* and *qph9-1*) mapped in our population has not been reported previously and may represent new genetic intervals linked to PH.

Previous studies have demonstrated that plant height, panicle length, heading date and flag leaf length present a significant positive correlation; multiple QTLs are mapped to the same area, such as plant height and spike length. This indicates that the relationship among the quantitative traits is extremely intricate; however, the functional direction does not change (Zhang et al.^2006^). This study revealed such cases: (Ping et al.^2003^) mapped *qph-2* between 5263536 and 30654749 bp on chromosome 2 with 81 *indica*-*japonica* DH populations. *qpl2-1* that controls panicle length in this paper falls within this interval; *qph3-1* and *qph3-2* mapped for PH characteristics that are linked to bin1846 (*qpl3-1*) and bin1943 (*qpl3-2*) for main PL when their positive alleles are derived from the Nipponbare parent with positive effects. There are also *qpl6-1* and *qpl6-2* with continuous peak values on chromosome 6. *qpl6-1* and *qph6-1 that* are mapped to the same bin (bin3619); *OsIAA23* (Jun et al.^2011^) is near to *qpl6-2* with peak value within the *ph6* interval (6927624-29906021 bp) (Figure 4B). The extended peak on chromosome 6 makes it possible that the new main QTLs exist within the genetic interval for PH and PL. When the remaining QTLs for main PL are compared with results from other studies, *qpl1-1* (LOD = 6.41) falls within the same interval as *qp1-1* mapped by (Hittalmani et al.^2002^). This locus was verified in *p11.1* in a BC_2_F_2_ population that was constructed from rice parents Jefferson and *O. rufipogon* (IRGC 105491) by (Thomson et al.^2003^). A locus distance that is more finely mapped than in previous studies is conducive to cloning this QTL; *qpl4-1* is similar to the locus interval mapped by (Zhuang et al.^1997^). *qpl10-1* on chromosome 10 was mapped to bin5745 while the dwarfing stem gene *brd2* (Hong et al.^2005^) falls within the area; the peak value of *qpl8-1* was mapped near to *IPA1* (Jiao et al.^2010^) for ideal plant architecture. *qpl12-1* was mapped to the long arm of chromosome 12, and this locus has not been reported yet.

GN per panicle, as a key constituent of rice yield, has been studied extensively. The main QTLs for genes that control GN are mapped or cloned accurately. In contrast to a previous study, a total of 11 genomic intervals associated with total GN per main panicle were mapped in this study, of which, three have been cloned. *qgn1-1* located on chromosome 1 and an allele of *Gn1a* (Ashikari et al.^2005^) from Habataki share the same region which became the dominant QTL for increasing the number of grains per panicle. The LOD score of *qgn1-1* in the population was 23.01, explaining 12.9% of the phenotypic variation; it can be seen from Figure 4C that chromosome 6 has a continuous peak, within which the methyl jasmonate (MeJA) biosynthesis gene *OsJMT1* (Kim et al.^2009^) is located at the same position as *qgn6-3*. The contribution rate of *qgn6-3* to GN per panicle is up to 8.9%; chromosome 8 has a prominent peak where *qgn8-1* is located. Subject to comparison, the locus has the same position as genes that control ideal plant type in *IPA1* for rice tillering, GN and 1000-grain weight. The next six QTLs (*qgn1-2, qgn2-1 qgn3-2, qgn6-1, qgn6-2* and *qgn6-4*) are similar to loci reported previously, with more accurate intervals. *qgn1-2* (*qLVBTS1-1* of Cui et al.), *qgn6-2* (*tns6* of Lin et al.) and *qgn6-4* (*gp6* of Hua et al.) have larger effect values (Cui et al.^2003^; Hua et al.^2002^; Lin et al.^1995^; Redona and Mackill^1998^; Yuan et al.^2003^; Zhuang et al.^2001^). These explain a total of 23.5% of the phenotypic variation, and provide a reference for rice GN breeding. However, we also found two new QTLs, *qgn3-1* and *qgn11-1* for GN.

The primary and secondary branches in rice panicles are important factors for determining GN. Studies have shown that the GN per panicle presents a significant or very significant positive correlation. Some super-hybrid rice varieties have giant panicles due to a larger number of primary and secondary branches and also have higher grain density (Wang and Li^2005^; Yang et al.^2000^). In this study, 16 QTLs linked to primary and SBNs were mapped; eight for each character. It can be seen from Figure 4D and E that chromosomes 1, 6, and 8 have loci with peaks for primary and SBNs. *qpbn1-1* for PBN and *qsbn1-1* for SBN were located in the same bin137 on chromosome 1 with QTL contribution rates of 3.3% and 14.1%. This is also the location of *Gn1a* for GN; *qpbn6-3*, *qsbn6-3* and *qgn6-4* were also mapped between the same intervals 36.5-36.8 cM. The locus is similar to *gp6* mapped by (Hua et al.^2002^) for GN; *IPA1* (Jiao et al.^2010^) located on the long arm of chromosome 8, which is a complicated gene that controls multiple yield traits in rice. In the present study, *qpbn8-1* for PBN, *qsbn8-1* for SBN and *qgn8-1* for GN are all close to *IPA1*. (Endo-Higashi and Izawa^2011^) grew four kinds of rice lines with different flowering stages under dissimilar photoperiods. The results showed that the *Hd1* and *Ehd1* genes can reduce the PBN, reduce the GN and control the dependent on the flowering stage. Hence, two key rice flowering genes, *Hd1* and *Ehd1*, control rice panicle development. Both loci affect the expression of the flowering locus in leaf, possibly having a further affect on rice yield in the field. Two QTLs, *qpbn6-2* and *qpbn10-1*, with larger effects, were mapped for PBN. Their LOD scores peak at 41.17 and 10.57 individually. Both intervals are located near to the genes *Hd1* (Yano et al.^2000^) for the heading stage and *Ehd1* (Doi et al.^2004^) for early heading. These QTLs explain 21.9% and 6.2% of the phenotypic variation separately, similar to their results. *qpbn6-1* mapped to chromosome 6 and *qNPB6-1* mapped by (Cui et al.^2002^) fall within the same interval when their effect values are larger among two dissimilar populations. *qpbn2-1* falls within *np2.2* (Marri et al.^2005^), and the regulon *OsFOR1* (Jang et al.^2003^) of rice flowering organs falls just within the *qpbn7-1* interval.

Additional QTLs for SBN are *qsbn1-2, qsbn6-1* and *qsbn6-2*, which fall within the same intervals as *qNSB1-1* (Cui et al.^2002^), *tns6* (Lin et al.^1995^) and *qSPN-6* (He et al.^2001^) respectively. *qsbn3-1* is located in the region for GN, which was mapped by (Redona and Mackill^1998^). *qsbn11-1* on chromosome 11 could be a novel genetic region that has not been reported previously.

Gene clustering is typically seen in preliminary QTL mapping studies. It signifies that QTLs for correlated traits are located in the same or proximate intervals on the same chromosome. Their positions are often close to one another, while QTLs that control dissimilar traits within the same interval and QTL that control the same trait in different intervals in the variation of genetic function mode, effect direction and effect size (Du et al.^2008^; Teng et al.^2002^; Zheng et al.^2003^). This is substantiated in QTL mapping in this study: within intervals of 5–25 Mb on chromosome 6, five yield factors (PH, PL, GN, PBNs and SBNs), appear as continuous peaks; a total of 14 QTLs were mapped, of which, four key genes, *OsIAA23, Hd1, Ehd1,* and *OsJMT1*, were directly mapped. We believe that QTLs within this interval will be resolved more accurately with the development of new genome sequencing technology and high-density SNP markers.

Six new intervals, *qph1-2, qph9-1, qpl12-1, qgn3-1, qgn11-1* and *qsbn11-1* are showed for the first time in this research. Among those, *qph9-1* and *qpl12-1* have lager effect values compared with the other. As the main yield related factors, we intend to develop CSSSLs (chromosome single segment substitution lines) or NILs (near-isogenic lines) of the two QTLs which should be useful to plant height and panicle type improvement. The other novel QTLs derived from hybrid rice elite parent R1128 which may play an important effect in super-yield rice breeding and could be used in rice pyramiding or MAS (marker assisted selection) breeding to increase the grain yields.

## Conclusions

Studies on rice functional genomics have been greatly facilitated by the use of high-throughput sequencing technology. In the present study, multiple sequencing was performed on progeny of an F_2_ population based on male parent R1128 and female parent Nipponbare. Multiple sequencing was preformed by using MSG sequencing technology, while QTLs linked to agronomic traits were mapped and analyzed for their effect. A total of 49 QTLs for five key yield factors, such as PH and PL et al., were mapped, and are distributed on 11 chromosomes (all except chromosome 5). A total of 14 QTLs were mapped on chromosome 6 alone; multiple major genes for good traits have been pyramided in R1128, including *Sd1* for plant height, *Hd1* and *Ehd1* for heading date, *Gn1a* for grain number and *IPA1* for ideal plant shape. These genes have independently explained 44.3%, 21.9%, 6.2%, 12.9% and 10.6% of the phenotypic variations of their traits. Six novel loci, *qph1-2, qph9-1, qpl12-1, qgn3-1, qgn11-1* and *qsbn11-1* are reported for the first time in this study. The super-hybrid rice parent R1128 is beneficial for rice breeding and the international resequencing varity Nipponbare has the clear genome background which is conducive to rice functional gene research. The genetic bin map constructed by R1128 and Nipponbare in this study is not only worth to rice gene fundamental research, but also valuable to practical application in rice breeding.

## Methods

### Plant material and phenotypic evaluation

In this research, we used 781 F_2_ (second filial generation) lines for the rice QTL mapping population. The population was developed from a cross between *Oryza sativa ssp. indica* cv*.* R1128 and *ssp. japonica* cv*.* Nipponbare followed by self-fertilization of the F_1_. The breeding of the *indica* restorer line R1128 was from a multiple cross between SH527 and an inbred F_4_ line which was generated from the cross between R855 and a temporary F_4_ line named 1033, which was introduced from America. The resulting F_1_ was crossed with the F_1_ from MH63 and R353 followed by selfing to the F12 (Liu et al.2012a). The *japonica* variety Nipponbare is the international sequenced cultivar (Goff et al.^2002^). All plant materials were cultivated at Changsha in China using normal field management practices.

An array of morphological characters including plant height (PH), grain number (GN), panicle length (PL), primary branch number (PBN), secondary branch number (SBN) of the two parental lines and the F_2_ population were investigated at Changsha in 2011. The examination standard was referred to (Zhao et al.^2007^) with little improvement.

### DNA isolation and MSG library preparation

Genomic DNA was extracted from small samples (0.5 g) of young leaves from the R1128 parent and the F_2_ progeny plants using CTAB. Whole genome re-sequencing was carried out for R1128 to varify its identification.

Genome-wide SNP development and genotyping for the F_2_ population were performed using MSG (multiplexed shotgun genotyping) as proposed by (Andolfatto et al.^2011^), with some modifications. Bar-coded adapters were designed and modified according to the standard Illumina adapter design for paired-end read libraries. Genomic DNA of each sample (1 μg) was digested with 1 μl FastDigest TaqI (Thermo scientific Fermentas) for 10 min at 65°C in a volume of 30 μl. Unique barcode adapters (10 μmol) were the added to each sample well. The ligation reaction was incubated 1 h at 22°C with T4 DNA ligase (Enzymatics) and heat inactivated at 65°C for 20 min. Twenty-four ligation products for different samples were pooled in a single tube and 2 μl chloroform was added to inactive the restriction enzyme. DNA fragments between 400–600 bp were then selected on a 2% agarose gel and purified using a QIAquick Gel Extraction Kit. All the products were amplified with 10 cycles of PCR (Phusion high-fidelity, Finnzymes) in a 50 μl reaction which included 25 μl Phusion Master Mix, 1 μl of common primer (10 μM) and 1 μl index primer. The amplified library was purified using a QIAquick PCR Purification Kit, quantified on the Agilent 2100 Bioanalyzer and finally sequenced on an Illumina Hiseq 2000 instrument.

### SNP identification

The rice reference genome from cultivar Nipponbare (IRGSPv6) was used to read mapping with the software SOAP2 (version 2.20) (Li et al.2009b). SOAPsnp (version 1.01) was used to generate the consensus sequences for each sample. Input data for SNP calling with realSFS (version 0.983) was prepared by SAMtools (version 0.1.8). Population SNP calling was performed with realSFS, based on the Bayesian estimation of site frequency at every site. The likelihoods of genotypes for each individual were integrated and sites with a probability of >0.95 and a population whole depth higher than 40 were extracted as candidate SNPs. Potential SNPs were then filtered using the following criteria: loci with >70% missing data that also showed serious distorted segregation of the two parental genotypes were excluded. All the SNPs were filtered using a PERL script.

The SNPs generated in this study were compared to the rice SNP database build by OryzaSNP Consortium (website: http://oryzasnp.plantbiology.msu.edu/). OryzaSNP Project SNP data download from http://ftp.plantbiology.msu.edu/pub/data/Oryza_SNP/. We use the SNPs identified by either the Perlegen SNP calls or the machine learning SNP calls as our reported SNP data set because it contains genomewide SNP variation for 20 diverse varieties and landraces that capture the impressive genotypic and phenotypic diversity of domesticated rice (Mcnally et al.^2009^).

For R1128, Indel calls were done with SOAPindel (version 1.08) and SV (structure variation) was identified with SOAPsv (version 1.02).

### Genotype calling and recombination breakpoint determination

We converted the SNP data into another format to simplify the genotype calling analysis. The SNP type from Nipponbare was coded as “a”, the R1128 alleles were coded as “b” and the heterozygotes were coded as “h”, while missing data was coded as “-“.

An F2 population that is temporary and collectively has SNPs in 50% heterozygous genotype, theoretically. The genotypes of the female and male parent genotype are dispersed irregularly in heterozygous regions. When SNPs detected from the F2 population were placed along the chromosomes, in a chromosomal region, SNPs representing one parent or both parents (heterozygous) were predominant and those representing the other parent were scattering among them. It is not accurate to determine the genotype of the F2 population based on individual SNPs. A sliding window approach adopted by Bin Han (Huang et al.^2009^) with some modification was used to evaluate a group of consecutive SNPs for genotyping. Firstly, based on the SNP density, we chose the window size of 15 SNPs for genotyping, which covered on average 75 kb or 0.3 cM of rice chromosomes. We also tested the effect of different window sizes on bin map construction and QTL analysis by using window sizes of 7, 11, 19 SNPs. The window sizes of 7, 11, and 19 yielded nearly identical results as the size of 15 in the identification of the largest QTL for plant height and grain number as the trait examples (Additional file7). Evidently, the higher sequencing coverage permits the use of larger windows covering the same physical and genetic intervals and consequently more accurate mapping, so we chose 15 SNPs as our analysis parameters. For each sample, a window of 15 SNPs without missing data was used for genotyping calling. An a/b ratio of 12:3 or higher was recognized as “a”, 3:12 or lower as “b” and anything in between as “h”.

We determined the breakpoints according to a published method for high-throughput genotyping by NGS (next-generation sequencing) with some modification (Davey et al.^2011^). Recombination breakpoints were determined by the junction of two different genotypes. For the breakpoints separating heterozygous from homozygous, which is a major kind of F_2_ population, we picked up the divert locus as recombination breakpoint. For heterozygous from homozygous, there were several temporary “h” and then changed into another genotype. The third changed locus was chosen for this breakpoint.

### Bin map construction and QTL analysis

According to the breakpoint information, we used a PERL script to generate bin information with intervals larger than 10 kb.

QTLs were identified by composite interval mapping using the software MapQTL5. QTL mapping in the present experiment was carried out by calculating the threshold logarithm of odds difference (LOD) for each trait by performing a test with 1,000 permutations. The experimental LOD threshold for every trait on each chromosome was calculated independently and the value could be at the 5% level of significance. QTL were named according to (Mccouch et al.^1997^) and the QTL mapping results were comprehensively compared to the OGRO (The Overview of functionally characterized Genes in Rice online database) (Yamamoto et al.^2012^), the Rice Genome Annotation Project, the IRGSP and Gramene data. The data for the population and parents for traits were calculated with SPSS statistics 17.0 (*P* < 0.05) and Microsoft Excel.

## Electronic supplementary material

Additional file 1: **The SNPs information generated from F2 population.** The documents including “chr01_filter.ab” to “chr12_filter.ab” are the samples genotype which converted to be a\b\h formats. It is marked “a” that the genotype of sample is the same to Nipponbare, or “b” represents the R1128 genotype and “h” is the heterozygous genotype; The deletion of sample gentoype is marked as “-” specially. Data files are generally TXT which compressed into a ZIP format. For Windows user,“Editplus” or “UltraEdit” is recommended as the browser program. Format description (left to right). 1. Chromosome. 2. Position. 3. Genotype of Nipponbare. 4. Genotype of R1128. 5. Genotype of sequencing sample. (ZIP 10 MB)

Additional file 2:**The SNPs information compared to the rice SNP database.** Format description (left to right). 1. SNP ID. 2. Chromosome. 3. Position. 4. Reference SNP. 5. Twenty diverse rice varieties and the last one is R1128. (ZIP 123 KB)

Additional file 3:**The genomic location of the breakpoints.** The physical position and genotype of the breakpoint are connected with “-”, such as “1140905-h”. Format description (left to right). 1. Individual sample on each chromosome. 2. The initial Genotype of Nipponbare. 3. Location and genotype of breakpoints of individual sample. (ZIP 289 KB)

Additional file 4:**Gentoype of sequencing samples correspond to each bin of chromosomes.** Format description (left to right). 1. Individual sample. 2. Genotype of single bin. (ZIP 121 KB)

Additional file 5: A genetic linkage map constructed with individual bin on chromosomes. (ZIP 270 KB)

Additional file 6:**The bin size and location for each bin. Format description (left to right).** 1. Chromosome. 2. Bin name. 3. The initial position of bin. 4. The terminational position of bin. 5. The size of bin. (ZIP 171 KB)

Additional file 7: Plant height and grain number QTLs detected on chromosomes when using different window sizes. (ZIP 251 KB)

Below are the links to the authors’ original submitted files for images.

Authors’ original file for figure 1

Authors’ original file for figure 2

Authors’ original file for figure 3

Authors’ original file for figure 4
