# Supplementary material for: Genetic analysis of an elite super-hybrid rice parent using high-density SNP markers
Source: Rice (N Y). 2013 Aug 15;6:21. doi: 10.1186/1939-8433-6-21 (PMC4883714; doi:10.1186/1939-8433-6-21)
Supplement: Supplementary file 5 — Additional file 5: A genetic linkage map constructed with individual bin on chromosomes. (ZIP 270 KB) [file 12284_2013_57_MOESM5_ESM.zip › Additional file 5//map1-6.pdf]

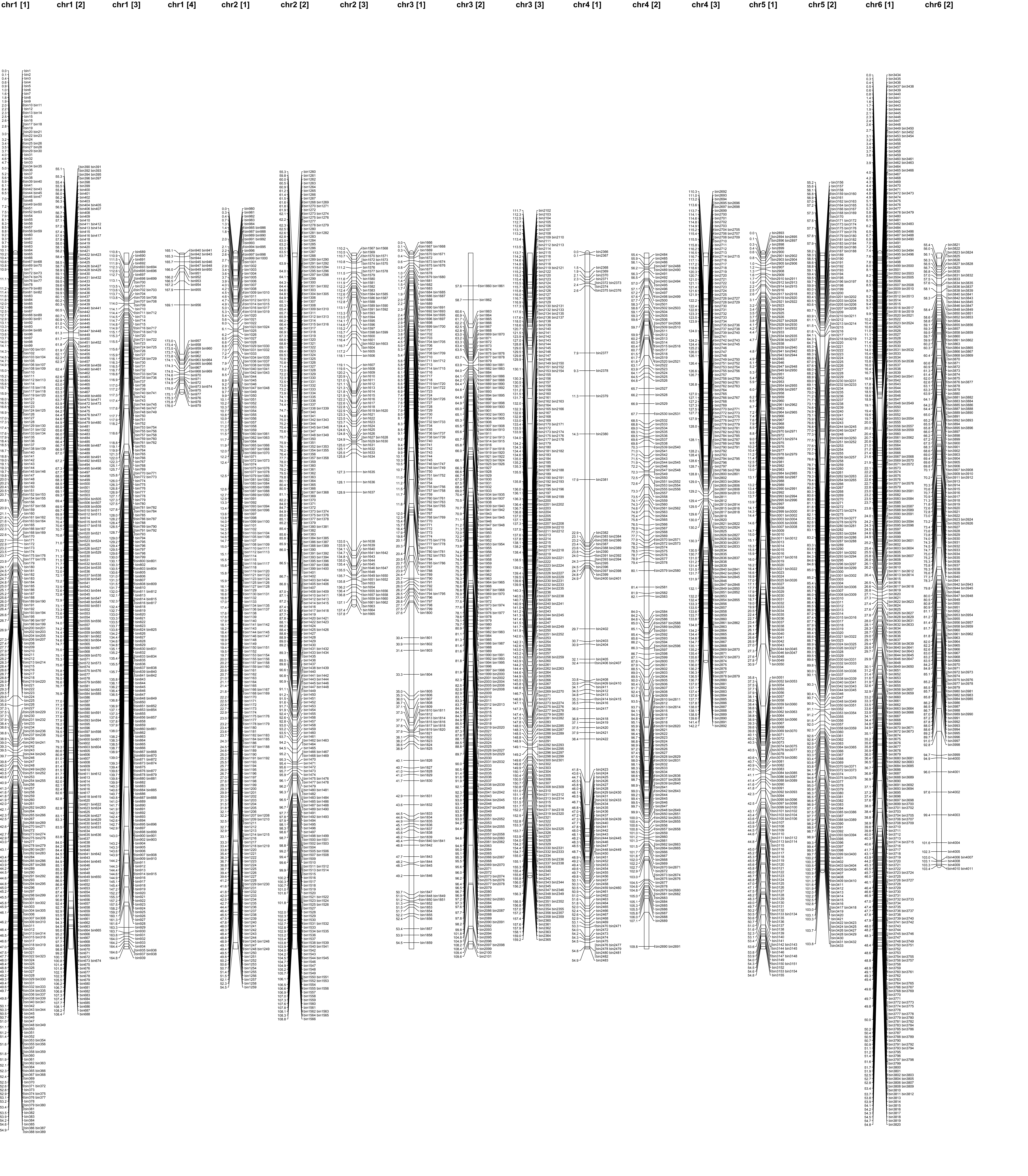
chr1 [1]
chr1 [2]
chr1 [3]
chr1 [4]
chr2 [1]
chr2 [2]
chr2 [3]
chr3 [1]
chr3 [2]
chr3 [3]
chr4 [1]
chr4 [2]
chr4 [3]
chr5 [1]
chr5 [2]
chr6 [1]
chr6 [2]
