# Supplementary material for: Genetic analysis of an elite super-hybrid rice parent using high-density SNP markers
Source: Rice (N Y). 2013 Aug 15;6:21. doi: 10.1186/1939-8433-6-21 (PMC4883714; doi:10.1186/1939-8433-6-21)
Supplement: Supplementary file 5 — Additional file 5: A genetic linkage map constructed with individual bin on chromosomes. (ZIP 270 KB) [file 12284_2013_57_MOESM5_ESM.zip › Additional file 5//map7-12.pdf]

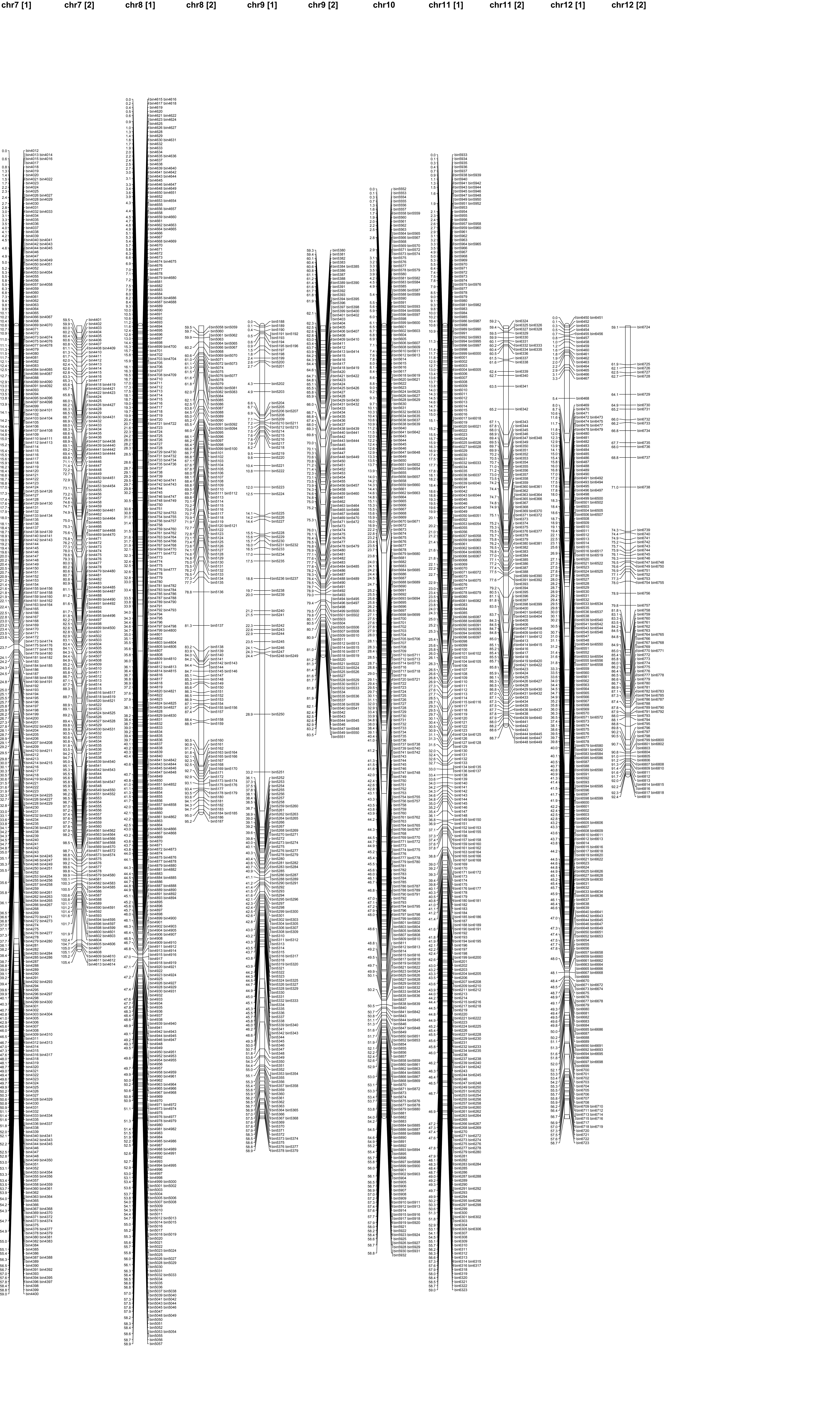
chr7 [1]
chr7 [2]
chr8 [1]
chr8 [2]
chr9 [1]
chr9 [2]
chr10
chr11 [1]
chr11 [2]
chr12 [1]
chr12 [2]
