# Supplementary figures and images for: Genetic analysis of an elite super-hybrid rice parent using high-density SNP markers
Source: Rice (N Y). 2013 Aug 15;6:21. doi: 10.1186/1939-8433-6-21 (PMC4883714; doi:10.1186/1939-8433-6-21)

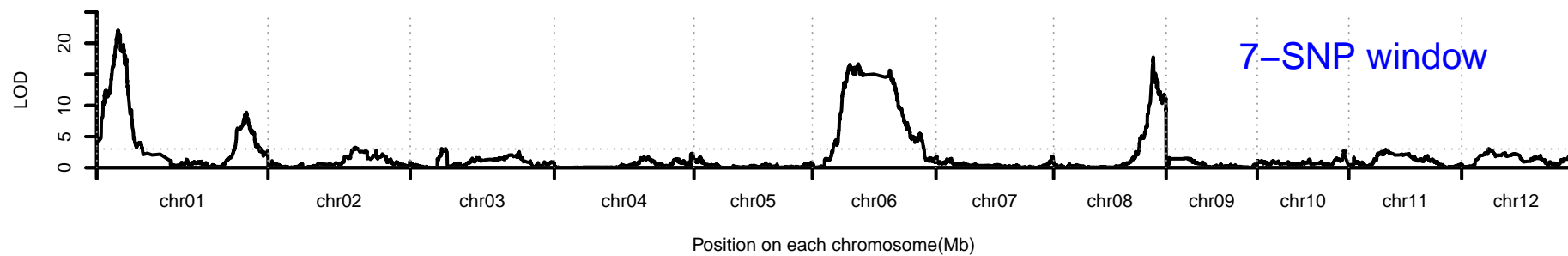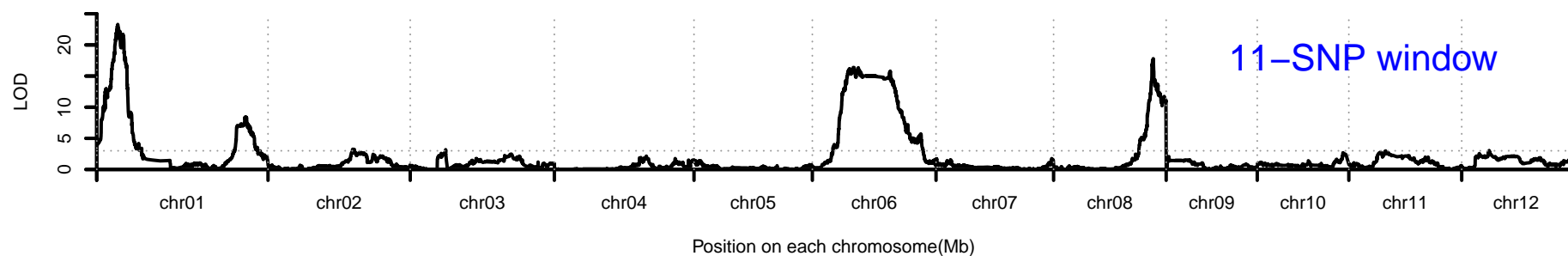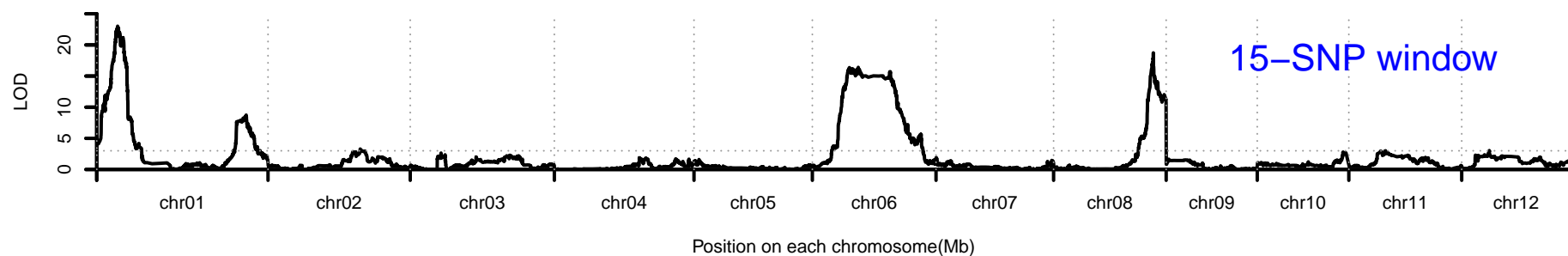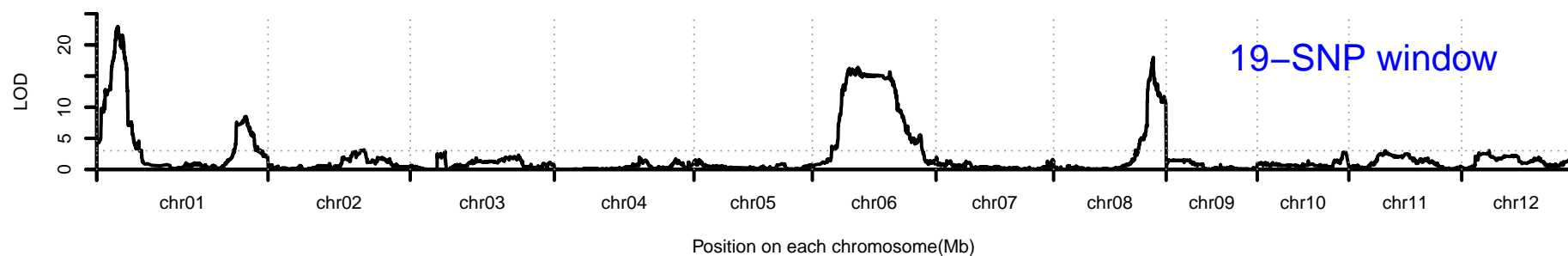

Supplement: Supplementary file 7 — Additional file 7: Plant height and grain number QTLs detected on chromosomes when using different window sizes. (ZIP 251 KB) [file 12284_2013_57_MOESM7_ESM.zip › Additional file 7//grain number.pdf]

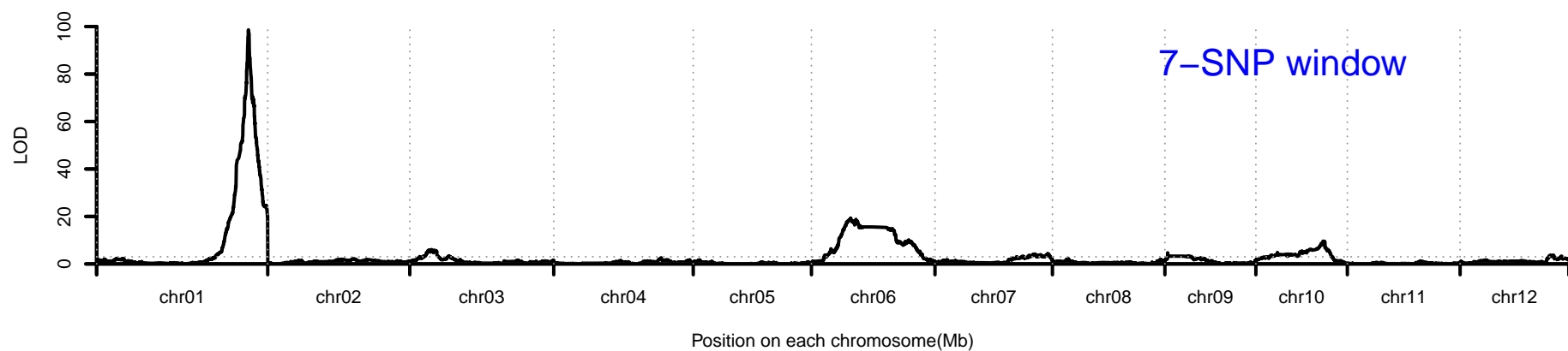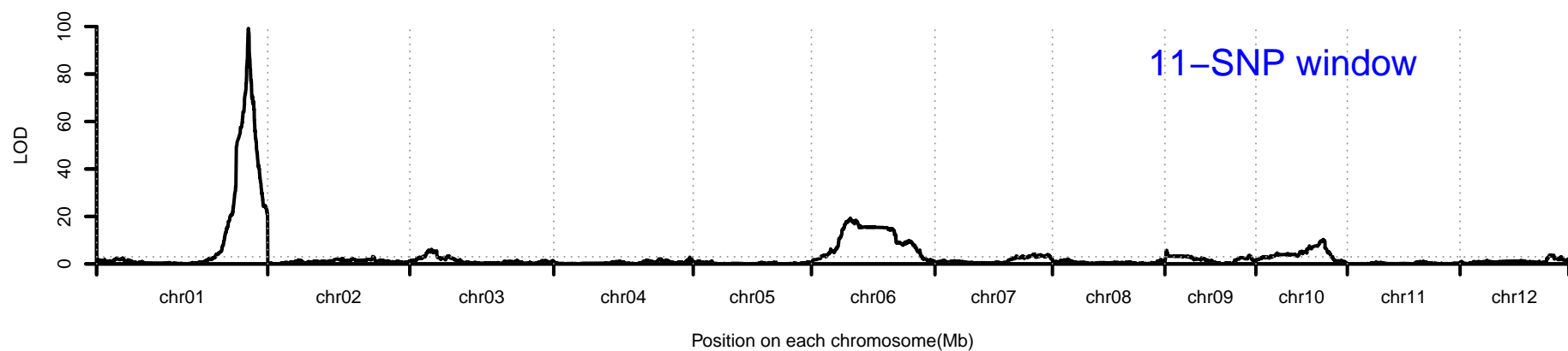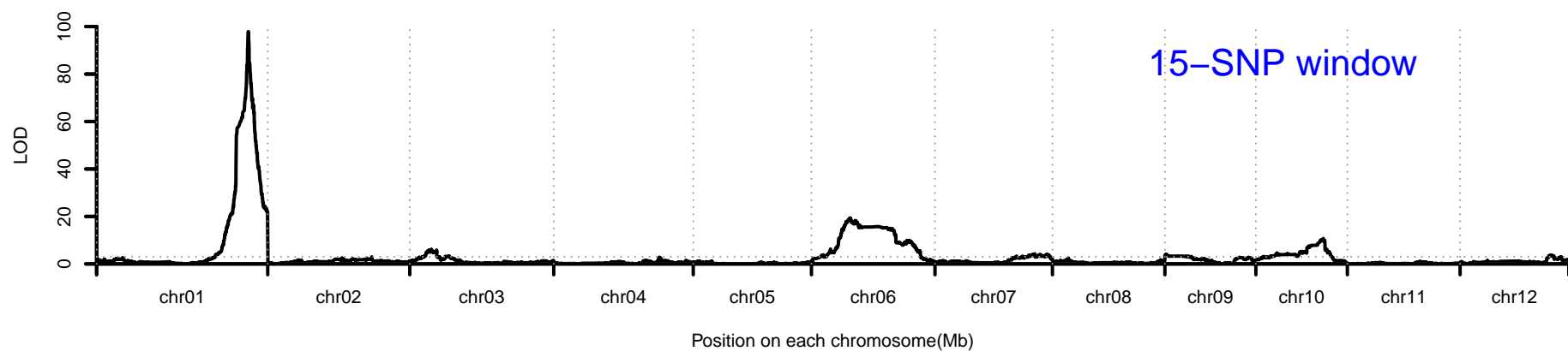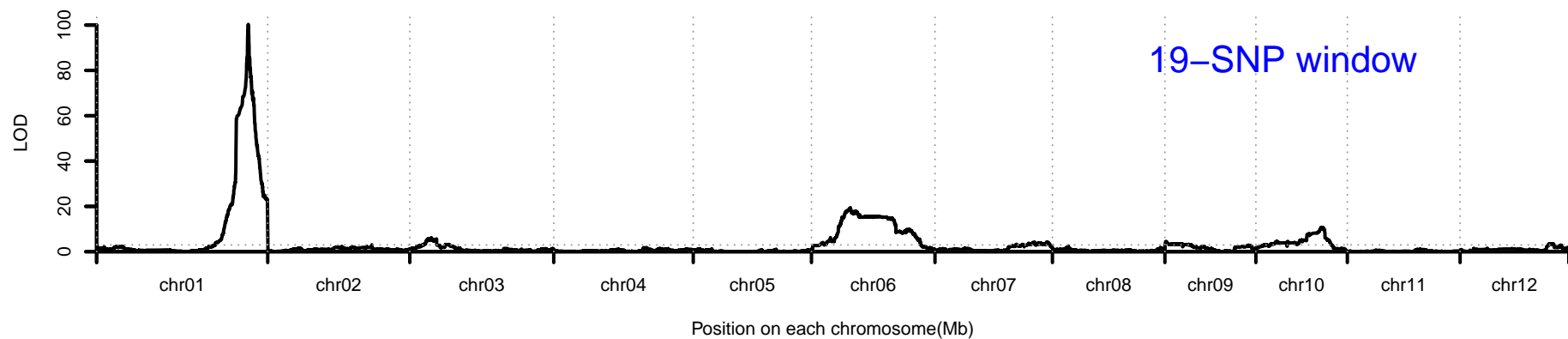

Supplement: Supplementary file 7 — Additional file 7: Plant height and grain number QTLs detected on chromosomes when using different window sizes. (ZIP 251 KB) [file 12284_2013_57_MOESM7_ESM.zip › Additional file 7//plant height.pdf]

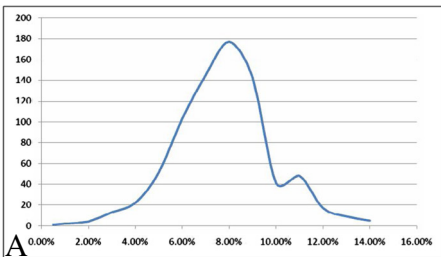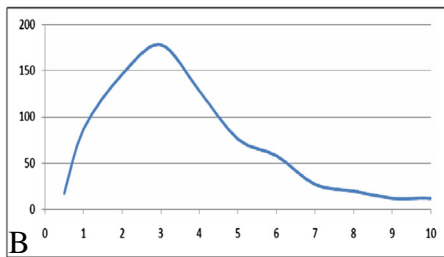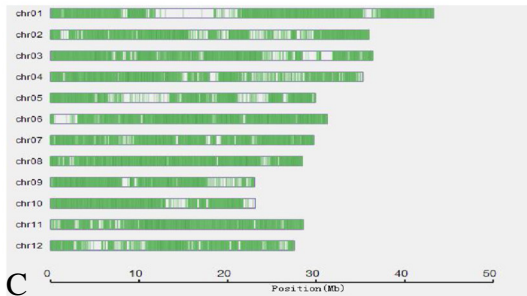

Supplement: Supplementary file 8 — Authors’ original file for figure 1 [file 12284_2013_57_MOESM8_ESM.pdf]

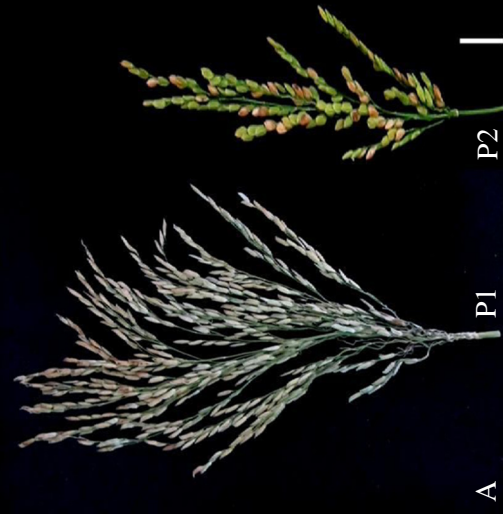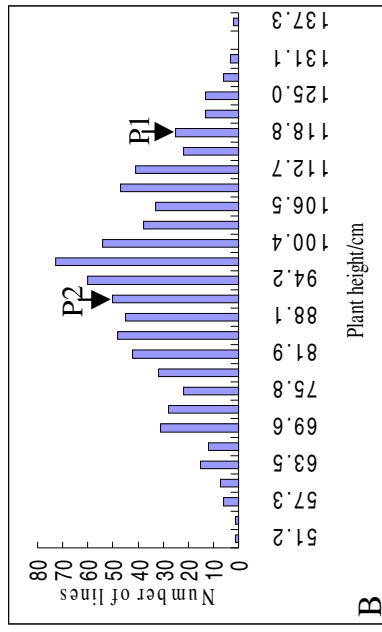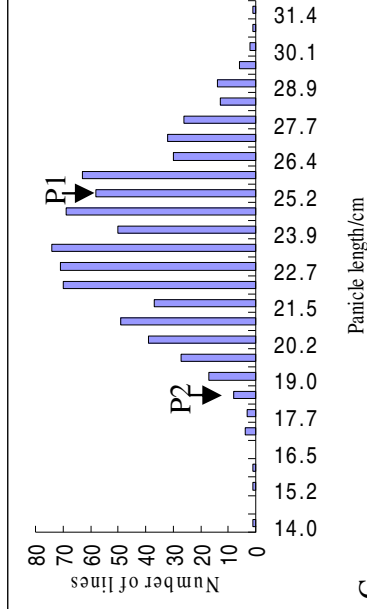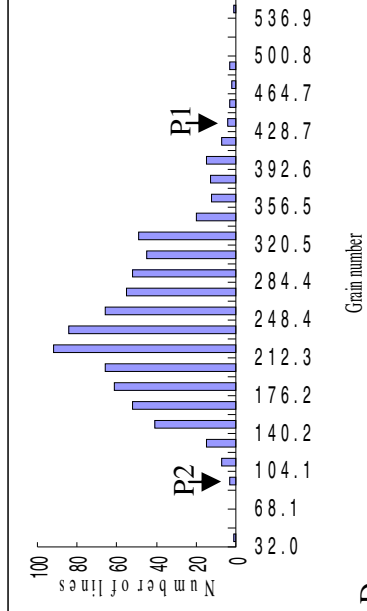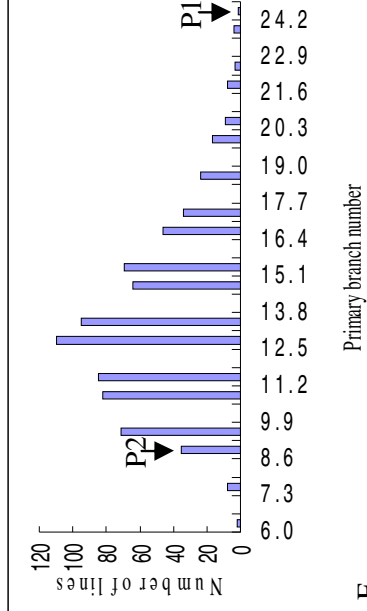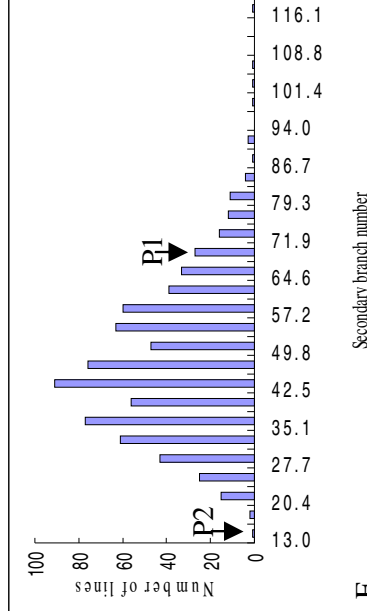

Supplement: Supplementary file 9 — Authors’ original file for figure 2 [file 12284_2013_57_MOESM9_ESM.pdf]

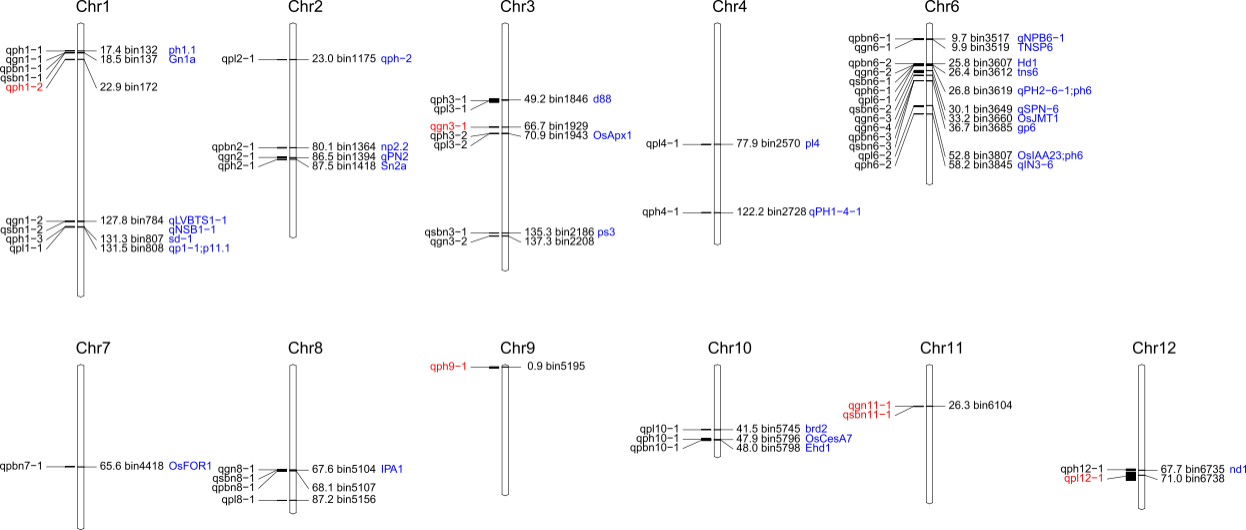

Supplement: Supplementary file 10 — Authors’ original file for figure 3 [file 12284_2013_57_MOESM10_ESM.pdf]

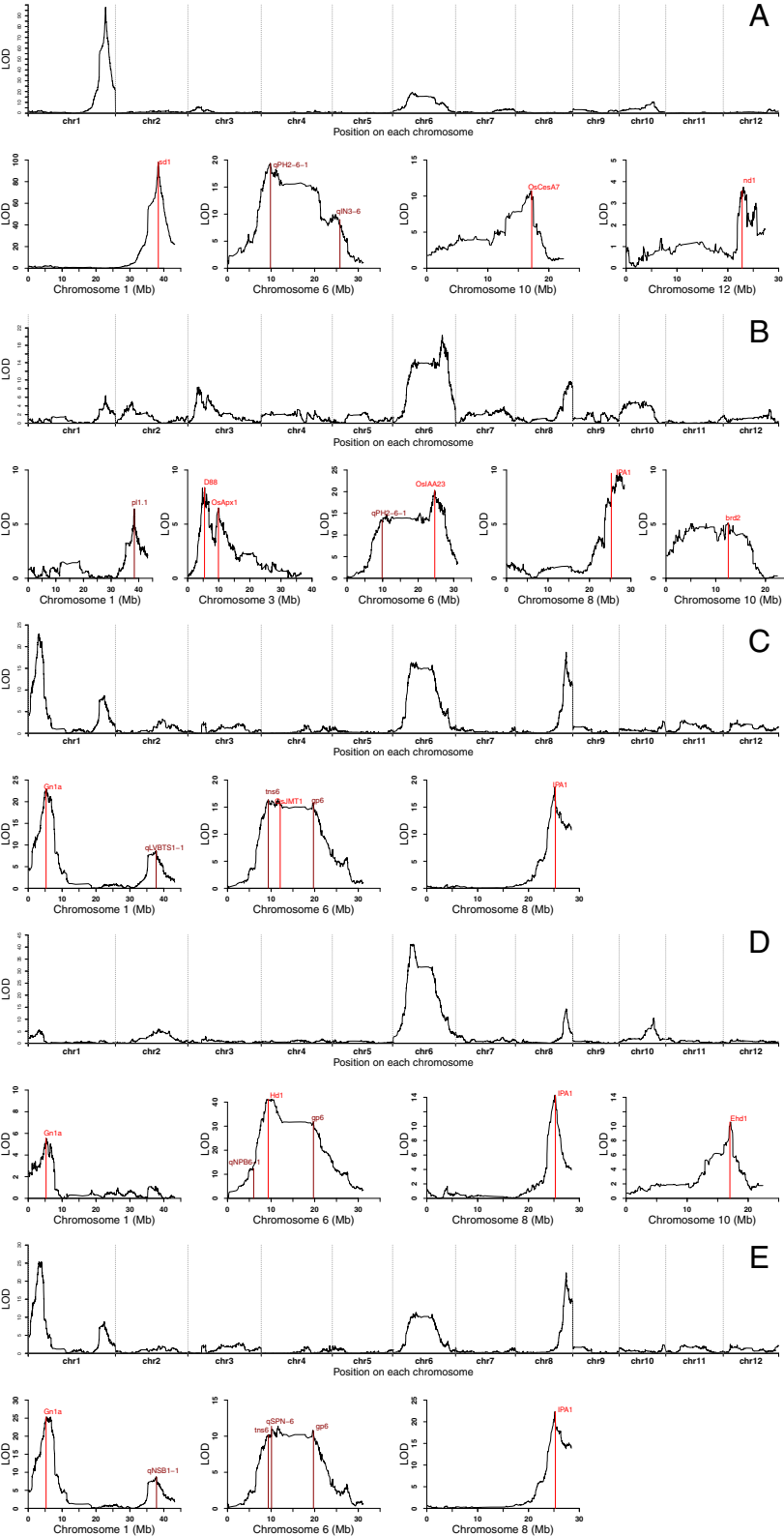

Supplement: Supplementary file 11 — Authors’ original file for figure 4 [file 12284_2013_57_MOESM11_ESM.pdf]
